# Potential Role of Cytochrome c and Tryptase in Psoriasis and Psoriatic Arthritis Pathogenesis: Focus on Resistance to Apoptosis and Oxidative Stress

**DOI:** 10.3389/fimmu.2018.02363

**Published:** 2018-10-30

**Authors:** Maria Sole Chimenti, Flavia Sunzini, Laura Fiorucci, Elisabetta Botti, Giulia Lavinia Fonti, Paola Conigliaro, Paola Triggianese, Luisa Costa, Francesco Caso, Alessandro Giunta, Maria Esposito, Luca Bianchi, Roberto Santucci, Roberto Perricone

**Affiliations:** ^1^Rheumatology, Allergology and Clinical Immunology, University of Rome Tor Vergata, Rome, Italy; ^2^Department of Clinical Sciences and Translational Medicine, University of Rome Tor Vergata, Rome, Italy; ^3^Dermatology, University of Rome Tor Vergata, Rome, Italy; ^4^Rheumatology Unit, Department of Clinical Medicine and Surgery, University of Naples Federico II, Naples, Italy

**Keywords:** psoriatic arthritis, psoriatic disease, autoimmunity, oxidative stress, apoptosis, cytochrome c and tryptase

## Abstract

Psoriasis (PsO) is an autoimmune disease characterized by keratinocyte proliferation, chronic inflammation and mast cell activation. Up to 42% of patients with PsO may present psoriatic arthritis (PsA). PsO and PsA share common pathophysiological mechanisms: keratinocytes and fibroblast-like synoviocytes are resistant to apoptosis: this is one of the mechanism facilitating their hyperplasic growth, and at joint level, the destruction of articular cartilage, and bone erosion and/or proliferation. Several clinical studies regarding diseases characterized by impairment of cell death, either due to apoptosis or necrosis, reported cytochrome c release from the mitochondria into the extracellular space and finally into the circulation. The presence of elevated cytochrome c levels in serum has been demonstrated in diseases as inflammatory arthritis, myocardial infarction and stroke, and liver diseases. Cytochrome c is a signaling molecule essential for apoptotic cell death released from mitochondria to the cytosol allowing the interaction with protease, as the apoptosis protease activation factor, which lead to the activation of factor-1 and procaspase 9. It has been demonstrated that this efflux from the mitochondria is crucial to start the intracellular signaling responsible for apoptosis, then to the activation of the inflammatory process. Another inflammatory marker, the tryptase, a trypsin-like serine protease produced by mast cells, is released during inflammation, leading to the activation of several immune cells through proteinase-activated receptor-2. In this review, we aimed at discussing the role played by cytochrome c and tryptase in PsO and PsA pathogenesis. To this purpose, we searched pathogenetic mechanisms in PUBMED database and review on oxidative stress, cytochrome c and tryptase and their potential role during inflammation in PsO and PsA. To this regard, the cytochrome c release into the extracellular space and tryptase may have a role in skin and joint inflammation.

## Introduction

**Figure 1:**
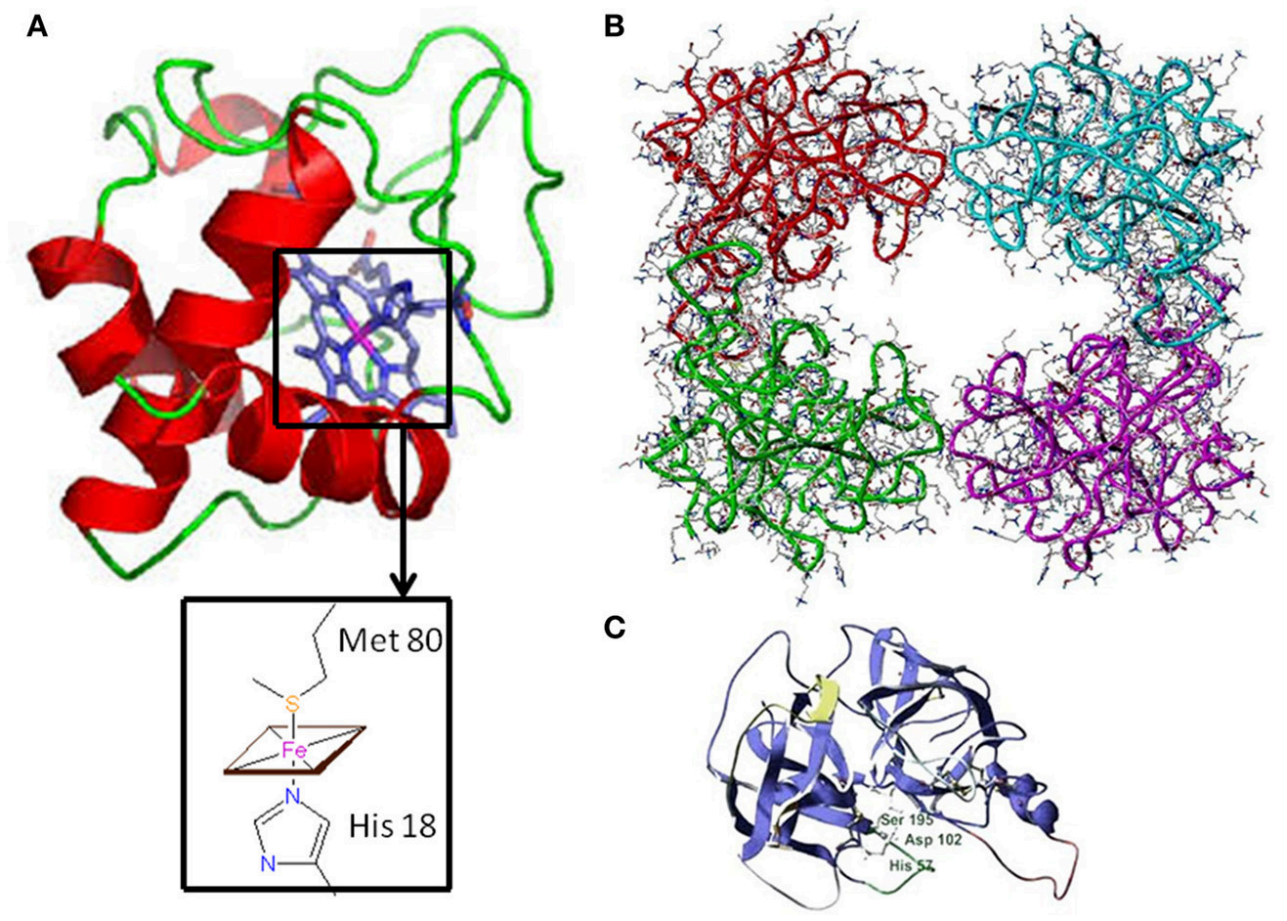
Three-dimensional structure of Cytochrome c and Tryptase **(A)** Horse heart cytochrome c structure characterized by X-ray crystallography (6). In the inset, the fifth and sixth ligands (His 18 and Met 80, respectively) to the Fe-Heme are shown. **(B)** Human β-tryptase tetramer structure as determined by X-ray crystallography (11). **(C)** Tryptase monomer structure: the catalytic triad residues (Ser 195, His 57, and Asp 102) are highlighted.

**Figure 2:**
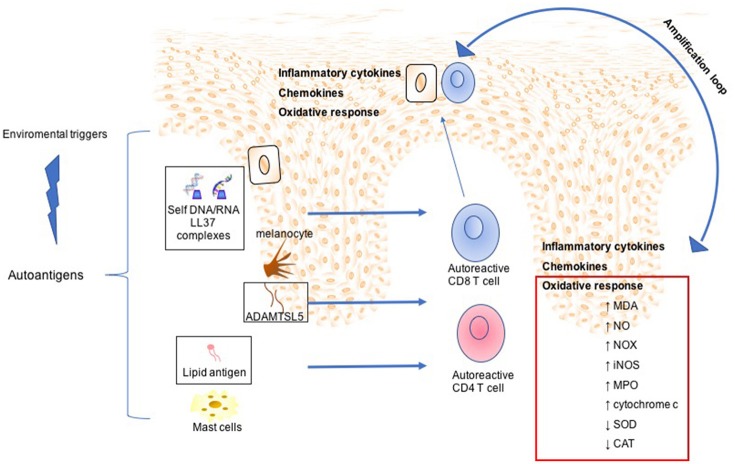
Mechanisms involved in Psoriasis arthritis pathogenesis. In a summary view, environmental triggers induce the expression of autoantigens, such as LL37 by keratinocytes, ADAMTSL5 by melanocytes, and lipid antigen by mast cells. This leads to expansion and activation of autoreactive CD8 and CD4 T cells. Although CD4 T cells remain within the dermis producing a large plethora of inflammatory cytokines, activated CD8 T cells migrate into the epidermis. Subsequently, potentially upon recognition of autoantigens in the epidermis CD8 T cells release other inflammatory mediators, which in turn mediate skin cell homing and remain as resident memory T cells in the epidermis. All this inflammatory process is maintained and amplified by oxidative stress sustained by production of NADPH-oxidase (NOX), inducible nitric oxide synthase (iNOS) myeloperoxidase (MPO), malondialdehyde (MDA), nitric oxide (NO), and serum cytochrome c and decreased of antioxidant status such as superoxide dismutase (SOD), catalase (CAT).

**Figure 3:**
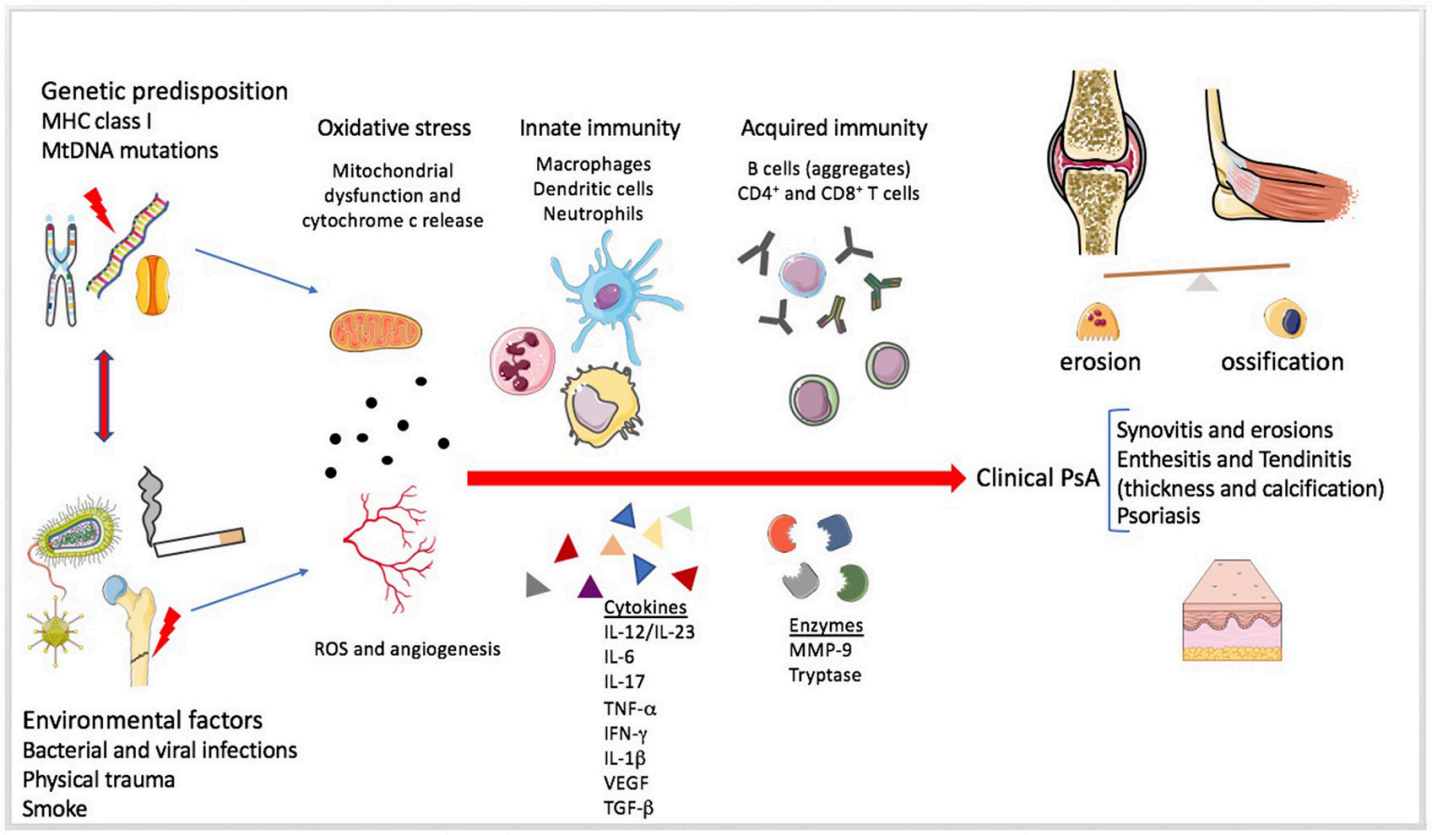
Mechanisms involved in Psoriatic arthritis pathogenesis. The figure summarizes the pathogenesis of Psoriatic arthritis (PsA). The enthesitis seems to be the primum movens of the disease, even if the heterogeneity of systemic involvement and clinical manifestations is extremely wide (31). Genetic and environmental factors predispose a healthy individual and contribute to the development of the disease. Mitochondrial dysfunction, angiogenesis and increased production of reactive oxygen species (ROS) seem to be present since the early disease onset. The role of the innate immunity in the tolerance disruption and in the production of a pro-inflammatory milieu is very early and essential in the pathogenesis of both PsA and Psoriasis. Adaptive immunity participates later by perpetuating and further increasing the inflammation. Several soluble mediators, such as pro-inflammatory cytokines and proteases, can be found in the synovial fluid and sera of PsA patients.

Psoriasis (PsO) is an inflammatory skin disease characterized by plaques of thickened and scaling skin due to keratinocyte proliferation, chronic inflammation linked to the presence of several innate and acquired immune cells, and mast cells activation (1). Psoriatic arthritis (PsA) is a chronic inflammatory arthritis that may be present in up to 42% of individuals affected by PsO (2). PsA is clinically characterized by inflammation of periarticular (e.g., enthesis) and articular structures. PsO and PsA share common pathophysiological mechanisms: the marked tortuosity of blood vessels and infiltration of plasma cell and mononuclear cells are observed in both the psoriatic plaque and PsA articular space (3). For both diseases, the pathogenesis is multi-factorial with underlying autoimmune mechanism (4). Genetic predisposition, as human leukocyte antigens (HLA)-Cw0602 and the HLAB27 allele, and an altered immune response can induce inflammation of skin and joints (5). Cytokines, as Tumor Necrosis Factor-alpha (TNF-α), interleukin (IL)-1β, IL-6, IL-17, and IL-18, are over-expressed in skin lesions, in peripheral blood, synovial membrane, and synovial fluid of PsA patients (6). Moreover, keratinocytes and fibroblast-like synoviocytes (FLS) exhibit similar resistance to apoptosis, one of the key mechanism that may facilitate psoriatic plaque growth and the hyperplasic progression of FLS in the synovium, destruction of articular cartilage and bone damage. PsA pathogenesis is only partially understood and the interest in the pathophysiological role of the synovium is recently growing (7). Pro-inflammatory mediators, responsible for joint inflammation, may be secreted at skin level and blocking this pathological communication may represent a new fascinating therapeutic objective. The primum movens seems to be the activation of the innate immunity. Several markers have been studied, among them, elevated S100A8 and S100A9 levels were observed in fluid samples from inflamed tissues in PsA synovium and in skin psoriasis in patients affected by both PsA and PsO (8). To this regard, inflammatory mediators, at an early stage, may predict articular involvement helping in preventing joint damage. Recently, the involvement of oxidative stress in PsO and PsA pathogenesis has been considered (9). Disruption of redox signaling, caused by oxidative stress, brings molecular damage which inevitably impacts on angiogenesis, inflammation, and function/activation of dendritic cells, lymphocytes, and keratinocytes (10). Cytochrome c, whose structure is shown in Figure 1A, is floating into the peripheral mitochondrial membrane and mediates the electron transfer (eT) throughout the respiratory chain (11–14). Reactive oxygen species (ROS) formation induces the release of cytochrome c into the cytosol (15, 16), where it binds to the apoptosis protease activation factor (APAf-1) forming with ATP or dATP, the apoptosome; this complex activates procaspase 9, which triggers an enzymatic cascade that brings to cell apoptosis (17–19). Several clinical studies regarding diseases characterized by cell death, either due to apoptosis or necrosis, reported cytochrome c release from the mitochondria into the extracellular space and finally into the circulation. Elevated cytochrome c levels in serum were found in chronic and acute diseases, including inflammatory arthritis, myocardial infarction and stroke, and liver diseases (20–22). Its role as an inducer of skin and joint inflammation has been proposed. In particular, a link between cytochrome c, skin inflammation, and keratinocytes proliferation was demonstrated during ROS production (23). ROS dysregulation and cytochrome c release have been associated with PsO, and were reported to be the cause of associated skin inflammation (24). During inflammation, another relevant pathway involves tryptase, a trypsin-like serine protease produced by mast cells and stored in intracellular vesicles. It is a catalytically active tetramer made of identical subunits, each having the catalytic triad residues (25) (shown in Figures 1B,C) and represents the major protein present in mast cells granules (26, 27). The tetrameric structure and the presence of heparin are necessary *in vivo* for the tryptase function (28). Tryptase activates, both *in vivo* and *in vitro*, a number of cells involved during innate and adaptive immune response, through proteinase-activated receptor-2 (PAR-2), thus contributing to the perpetuation of inflammation (29). The activation of PAR-2 brings about, in some circumstances, apoptosis inhibition (30). Several new interesting pathogenetic ways have been recently assumed in Psoriatic disease pathogenesis (23, 25). This review aims at discussing the role played by two new inflammatory mediators, as cytochrome c and as tryptase, in regulating extrinsic and intrinsic apoptotic pathways in PsO and PsA pathogenesis (as described in Figures 2, 3). The role of oxidative stress, linked to psoriatic disease pathogenesis, will be highlightened. Moreover, the review will focus on the relationship of these two molecules with oxidative stress and their potential role as inflammatory markers in PsO and PsA.

## Potential role of cytochrome c and tryptase in psoriasis and psoriatic arthritis

### Cytochrome c, apoptosis and chronic inflammation

Cytochrome c was found to be one of the key signaling molecules during programmed cell death-apoptosis. As explained above, its translocation into the cytoplasm leads to its interaction with APAf-1 and, in presence of ATP/dATP, to the final activation of procaspase 9. These stages are crucial for the completion of the apoptosis (16). In normal conditions, the apoptosis leads to cells death in dysfunctional cells owing to the strictly regulated pathways which are vital to maintain the homeostasis. A disequilibrium in the regulation of the apoptotic way may lead to defective immune responses and, consequently, to infections, tumor growth and autoimmune diseases. There are two intracellular pathways leading to apoptosis: the extrinsic, mediated by death-receptors, and the intrinsic or mitochondrial pathway (32). ROS are known to cause mitochondrial damage and dysfunction, which can cause the rise of cytochrome c release into the cytoplasm, as part of the intrinsic pathway, and the activation of the apoptotic pathway (33). In this context, it has been demonstrated that an increase in the release of cytochrome c from the mitochondria into the cytosol, is regulated by B-cells follicular lymphoma (Bcl)-2 family proteins (33). Therefore, the regulatory effect of the Bcl-2 family is essential for the control of apoptosis and can determine the cell's fate (33). Schultz and colleagues described how deregulation of apoptosis, linked to the intrinsic pathway, may contribute to autoimmunity (34). They observed that mainly Fas ligand (FasL) contributes in the pathogenesis of autoimmune diseases. FasL and other (TNF) ligands bind death receptors and induce: the release of cytochrome c and the expression of both flavoprotein apoptosis-inducing factor and the second mitochondria-derived activator of caspases, known as DIABLO (inhibitor of anti-apoptotic factors and activator of procaspase) (34). The consequence is the activation of caspases with the induction of cell death through DNA and protein cleavage. Cellular damage may release potential autoantigens and may be the basis for autoimmunity and inflammation.

During autoimmune diseases, the regulation of self-reactive B cells, in order to inhibit autoantibody production, may be critical. B cells' tolerance consists in the inhibition of completely or partially autoreactive B cells by using different mechanisms. Cell death pathways are the most reliable system to eliminate autoreactive B cells and to prevent autoimmunity (35). The presence of autoreactive cells and mitochondrial markers, as cytochrome c, may be relevant also in PsO and PsA pathogenesis (22, 36). In PsO, a dysregulation of cytochrome c has been demonstrated. Indeed, a higher serum and mitochondrial levels were observed compared to healthy skin; this contributes to epidermal hyperplasia (37). Concerning PsA, the result is synovial inflammation and joint destruction (38). PsA is microscopically characterized by lining layer hyperplasia, infiltration of B and T lymphocytes, and activation of the innate immune response that lead to vascular remodeling and angiogenesis into the joint (39). The characteristic PsA synovitis has inflammatory infiltration of macrophages, lymphocytes, and plasma cells that cause FLS hyperplasia and chronically, joint and bone damage (40). A cytochrome c dysregulation may also result in an inadequate apoptosis. To this regard, both the FLS and keratinocytes display a resistance to apoptosis leading to synovial and epidermal hyperplasia which are the hallmark of synovitis and psoriatic plaques genesis (41, 42). In this context, impaired apoptosis may contribute to the perpetuation of the inflammatory process (43). For this apoptotic resistant state, Mitomycin C (MMC), which is a bacteric anti-tumor antibiotic, has been investigated as potential treatment in inflammatory arthritis. This agent has shown an inhibitory effect on fibroblast proliferation by inducing apoptosis (39). Furthermore, Yan and coll. have demonstrated that MMC can decrease cell liability and provoke apoptosis in FLS obtained from patients affected by inflammatory arthritis, as rheumatoid arthritis (RA). A possible mechanism involved in the MMC inhibitory effect on FLS may regard the intrinsic mitochondrial pathway. To this regard, MMC stimulates the production of ROS and induces the release of cytochrome c, and consequently it may activate the caspases' cascade (33).

### Tryptase and chronic inflammation

Among cells that participate to the inflammatory status, the role of mast cells during inflammation was well demonstrated in the literature (44). Mast cells activation into joints and skin can cause the release of vasoactive mediators, such as histamine, prostanoids, and cytokines which contribute to inflammation (44, 45). Moreover, tryptase, released by mast cells, play an important role in the pathogenesis of inflammatory arthritis (46). Different pathogenetic mechanisms are linked to tryptase release: it is expressed in high concentration in synovial fluids from patients affected by RA, PsA, and reactive arthritis (47). The link between PsA and tryptase is supported also by the presence of corticotropin releasing hormone receptor 1 in PsA synovial biopsy. This hormone and its receptor have been demonstrated in the joints of patients affected by PsA and not in the control group. This hormone can enhance the intercellular matrix degradation by inducing tryptase release by mast cells (48). Intriguingly, a mast cell subset able to produce IL-17 was detected in rheumatoid synovium (49) and in psoriasis plaques, resulting to be the prevalent IL-17 producing cells (50, 51). The relevant role of IL-17 in the pathogenesis of PsO and PsA is pointed out also by the efficacy of biological drugs as IL-17 inhibitors for both skin and joint involvement in these diseases (52–55). To note, tryptase showed to have several pathogenic roles in PsO and PsA patients: (1) it can induce the production and release of several pro-inflammatory cytokines, such as IL-1β, IL-8, TNFα, and IL-6 (56, 57); (2) it stimulates leukocytes migration into the joints by PAR-2, ICAM-1, CXCR2, and IL-8, directly causing and amplifying the inflammatory process (58–60); (3) it activates, by cleavage, proteolytic enzymes, such as prostromelysin, procollagenase, and MMP-3 which contributes to matrix degradation (61, 62). Furthermore, it has been demonstrated that mast cells proteinases, as tryptase, secreted in excess under the influence of inflammatory stimuli of specific cytokines, degrade the joint matrix *in vitro*. Tryptase damages various matrix components by the activation of matrix metalloproteinases (62). Mast cells can express several cytokines, as TNF-α and IL-1β, and various profibrotic cytokines such as TGF-α and IL-4, suggesting numerous functional roles for Mast cells during chronic inflammation (63). In particular, synovial mast cells *in vitro* release tryptase and activate latent collagenase (64), participating to the development of the typical PsA synovial hypertrophy (65).

## Evidence for autoimmune pathways and oxidative stress in psoriasis

### The autoimmune side of psoriasis pathogenesis

PsO is immune-mediated inflammatory cutaneous disease characterized by keratinocyte proliferation and chronic inflammation (58). Recently, evidence has supported that PsO has an autoimmune pathogenesis and three different autoantigens have been identified so far: cathelicidin LL37, a domain thrombospondin type 1 motif-like 5 (ADAMTSL5) present in metalloproteases, and lipid antigens generated by phospholipase called PLA2G4D (66, 67). LL37 is a peptide upregulated in psoriatic skin with antimicrobial properties, LL37 can bind self RNA and DNA in complexes which are able to activate plasmacytoid and myeloid dendritic cells (68). This leads to an expansion of LL37-specific T cells producing pathogenic cytokines such as INF-γ, IL17, and IL-22. LL37 presentation to CD8 and CD4 T cells is mediated by HLA-Class I, in particular (HLA)-Cw0602 and HLA-Class II molecules respectively (69). ADAMTSL5 is an autoantigen presented by (HLA)-Cw0602 in PsO, the derived peptide can stimulate psoriatic T cells but not T cells from healthy individuals, resulting in IL17 production (68). Moreover, ADAMTSL5 and LL37 are increased in psoriatic lesions and are co-expressed by many immune cells, dendritic cells, neutrophils, macrophages, and T cells within skin. Both ADAMTSL5 and LL37 can be decreased by treatment with IL-17 or TNF-α inhibitors (70). Lastly, there is evidence for non-peptide autoantigen in PsO. T cells from PsA patients can recognize also lipid antigens generated in mast cells by PLA2G4D which are presented by CD1a. PLA2G4D is expressed in psoriatic skin lesions, but not in skin of healthy individuals (71). PsO pathogenesis is multifactorial, resulting from a combination of genetic, epigenetic, and environmental factors which lead to activation of an abnormal immune response. Working models for PsO suggest that several immune cells may present these antigens to autoreactive T cells with following activation and clonal expansion (60). This mechanism induces cytokines production, immune cells activation, and cell recruitment which in turn contributes to the amplification of inflammatory response and keratinocytes proliferation in PsO.

### The role of oxidative stress in psoriasis pathogenesis

In this complex pathogenesis, oxidative stress and free radical production play a role in skin inflammation (23). It was demonstrated that a reduction of antioxidant and augmented oxidant activities in psoriasis exists (72, 73). Moreover, a reciprocal amplification loop may exist between inflammation and oxidative stress in PsO. It is known that ROS production during oxidative stress activates cellular proinflammatory signaling mostly the JAK–STAT, MAPK/AP-1, and NF-κB pathways, leading to the production of cytokines, chemokines, and growth factors which are involved in the pathogenesis of psoriasis (74–77). Interestingly, activator protein-1 (AP-1) activates peroxisome proliferator-activated receptor δ (PPARδ) which is up-regulated in PsO, inducing proliferation and preventing apoptosis of keratinocytes, via the activation of heparin-binding EGF-like growth factor (HB-EGF) and activation of Protein Kinase Ba/Akt1 pathway, respectively (78, 79).

On the other hand, oxidative stress may originate by endogenous stimuli such as Th1 and Th17 cytokines, which are able to induce the production of NADPH-oxidase (NOX), inducible nitric oxide synthase (iNOS) and myeloperoxidase (MPO) and from environmental agents that may be also the cause of inflammation (80). Moreover, it has been recently demonstrated that MPO can be considered as a marker of systemic inflammation in Psoriatic disease (81).

Gabr and colleagues demonstrated a positive correlation between serum malondialdehyde, nitric oxide and serum cytochrome c levels and disease severity (measured by PASI score). On the contrary, a negative correlation with superoxide dismutase (SOD), catalase, and total antioxidant status was reported (36). Increased levels of mitochondria cytochrome c were also observed in lesional psoriatic skin compared to non-affected skin, and this increase is reversed by methotrexate (MTX) treatment (37). Indeed, during MTX therapy, an increased cytosolic cytochrome c level and consequent cleaved caspase-9 were also observed (37). Apoptotic dysregulation has been reported in psoriatic keratinocytes, which display a resistance to apoptosis, contributing to epidermal hyperplasia (42). Accordingly, in a recent article, after UVB irradiation, psoriatic keratinocytes showed a less cytosolic cytochrome c level compared to keratinocytes from healthy skin (82). A wide type of cells, such as T cells, dendritic cells, neutrophils, keratinocytes, mast cells, NK cells and macrophages are involved in PsO pathogenesis with different roles and specific hallmarks (83). The activation of innate immune system is believed to play a key role in the initial step of plaque formation, and, in this context, mast cells contribute producing IL-22 and IL-17 (52, 84). Furthermore, a high number of mast cells are present in the affected skin as well as the associated release of tryptase and histamine (85). Tryptase positive mast cells are increased in psoriatic lesional skin compared to normal skin, and tryptase mainly is localized in the dermis, at dermis-epidermal junction and around blood vessels, as showed by Steinhoff and collaborators through immunohistochemistry experiments (45). Tryptase participates to PsO inflammation through specific cytokines and neuropeptides production. Indeed, Tryptase acts by PAR2 cleavage, which is highly expressed in mast cells and in keratinocytes of PsO skin lesions (86–88). PAR2 exerts its inflammatory effects inducing the production of proinflammatory cytokines such as TNFα, IL-1ß, IL-6, IL-8, and granulocytes macrophage-colony stimulation factor (GM-CSF) (57, 87, 89). In details, TNFα is a landmark inflammatory mediator in PsO, being one of the first target of monoclonal antibody therapy (90) and GM-CSF is a mediator for maturation of Langerhans cells and stimulates keratinocytes proliferation (91, 92). On the other hand IL-6, which is also highly expressed in PsO, is able to stimulate keratinocytes proliferation (93) and contributes to the differentiation of Th17 cells which play a key role in PsO pathogenesis (94). Interestingly, PAR2 is also able to induce skin inflammation also through neurogenic mechanism, stimulating sensory C-fibers to produce some neuropeptides such as calcitonin gene-related peptide and substance P (95).

## Role of autoimmunity and oxidative stress in psoriatic arthritis

### The autoimmune side of psoriatic arthritis pathogenesis

The autoimmune side of PsA pathogenesis has been demonstrated by the presence of autoreactive T cells in synovium from PsA patients, which were activated by a homologous protein antigen, expressed in the synovium (4). Furthermore, the association with major histocompatibility complex class I molecules, the loci HLA-Cw6 or HLA-B27 in particular, leads to the presentation by those molecules of an autoantigen with the consequent production of antibodies against autoantigens. Additionally, the good response to immunosuppressive agents and cytokines blockers supports the involvement of autoreactive cells participating at the autoimmune process (96). Histologically, the PsA synovium presented both clonal and non-clonal T cells (97), and the link between skin and synovium T cells-clones (98) has been demonstrated. This indicates that a common antigen might drive the T cell response in both the target organs. Concerning the joint-enthesal complex, a group of cross-reactive PsA-specific antibodies, directed against peptides expressed in both the psoriatic skin and the inflamed enthesis, has been demonstrated by Dolcino and collaborators (99). Moreover, recent evidence suggests that PsA synovial biology involves several type of immune cells. The same histological heterogeneity observed in RA synovium has also been detected in psoriatic synovial tissue (100). In particular, lymphocytes infiltrates, in PsA synovium, tend to aggregate in lymphoid agglomerates, supporting the presence of an ectopic lymphoid neogenesis (LN), and autoantibody production (100). Conigliaro et al. previously reported an abnormal distribution of peripheral blood B cells in both RA and PsA patients (101). Peripheral B cells were reduced in PsA patients and their level were restored after anti-TNF treatment suggesting a role of B cells in PsA pathogenesis (101). During inflammation, several protein modifications may occur supporting the strong link between autoimmunity and inflammation. Among them, the effects of carbamylation on proteins and its effect in inflammatory arthritis has been recently investigated. Carbamylation is a post transcriptional modification on lysine, the effect on protein function and biochemical properties includes a change of the ability in polymerization, sensitivity to proteinases, and binding avidity to antibodies (102). To date, it is well known that carbamylation happens during inflammation, after the release of MPO by neutrophils, generating a pro-inflammatory loop. Carbamylated proteins are recognized by circulating antibodies, the anti-carbamylated protein (anti-CarP) antibodies that have been identified in patients with RA, also before the clinical onset of the disease (38). The presence of anti-CarP antibodies in sera from PsA patients with active disease, in the absence of rheumatoid factor and/or other known autoantibodies specificities, was demonstrated (96, 102). Interestingly, the circulating anti-CarP antibodies levels showed to be a good diagnostic test for PsA patients in comparison to healthy subject. A further support of an autoimmune origin is the discovery of a peptide antigen, called the PsA antigen, recognized by IgG derived from PsA patients' sera. This peptide shows homologies with peptides expressed both in skin and enthesis, as a further demonstration that an immunologic disequilibrium has a role in the pathogenesis of PsA (99).

### The role of oxidative stress in psoriatic arthritis pathogenesis

The activation of innate immunity is believed to have a role in PsA pathogenesis (96). In particular, the monocyte/macrophages population has a relevant role during inflammation at the enthesal level: it participates to the tolerance disruption in PsA patients and activates the release of mediators linked to the oxidative stress (103, 104). The oxidative damage in PsA is mainly due to a dramatic production of ROS that saturate the compensatory antioxidant enzymes and molecules, such as glutathione and SOD (105). The substantial producers of ROS are present in the mitochondrial membrane, NADPH oxidase and eT chain. The consequence of the unbalanced production of ROS is the leakage of cytochrome c from mitochondria, which activates the caspases cascade leading to apoptotic cell death (105). This reaction occurs also in cells populating inflamed joints, like synoviocytes, chondrocytes, lymphocytes and monocytes. To note, oxidative stress can lead to cyclooxygenase-2 and MMP9/13 expression and modulates apoptotic pathways and NF-κB. All of them are relevant mediators during inflammatory arthritis pathogenesis (106–109). Recent studies have highlighted the possible role of oxidative stress in PsA. Altered levels of oxidative metabolites, for example carbonyl groups, F2-isoprostanes, hydroperoxides, and sulfhydryl groups, were detected in PsA patients when compared to healthy donors (110–112). A similar study, showed a higher serum peroxide concentration and a lower antioxidant capacity in sera from patients affected by RA, PsA, and PsO in comparison with healthy individuals (113). The connection between circulating ROS and inflammation was investigated in RA, PsA, and ankylosing spondylitis patients in whom ROS in sera were higher when compared to controls (113, 114). Interestingly, after anti-TNFα treatment, a reduced value of circulating ROS was detected (114). Furthermore, the circulating peripheral blood mononuclear cells in patients with inflammatory arthritis have high levels of peroxidated lipid and altered polarization of the mitochondria membrane; these alterations correlate with patients disease activity (115). Furthermore, oxidative stress takes part also in the pathogenesis of comorbidities that may affect PsA patients. Patients with PsA have an accelerated atherosclerosis, and so a higher cardiovascular risk compared to healthy subjects (116). In this context, oxidative stress plays a relevant role in the pathogenesis of atherosclerosis: it induces the oxidative modification of LDL. The oxidized LDL takes part in many phases of atherogenesis and are strictly related to the inflammatory process (117). Moreover, oxidative modifications of LDL have been linked to the presence of TNFα and HDL may be altered during inflammation. Modified HDL lose their capacity to remove cholesterol from atherosclerotic lesions and have a reduced antioxidant activity (118). During inflammation, the release of TNFα is one of the main contributor of increased ROS production, and this is strongly related to disease activity (119). Then, the presence of ROS supports oxidative stress, further stimulating in this way cell damage and atherogenesis (113). Recent findings demonstrated that ROS were found not only into the blood stream and in circulating cells, but also into the joint. In synovial fluid of PsA patients, a higher ROS production, angiogenesis, and, DNA damage was found when compared to an osteoarthritis group (120–122); this is a further demonstration that oxidative stress has a role in the pathogenesis. Interestingly, a role of hypoxia was proven during inflammatory arthritis pathogenesis. A negative correlation between pO2 in synovial fluid of RA and PsA patients and level of macroscopic synovitis, disease activity, sublining layer thickness, and cells infiltration were demonstrated. In addition, hypoxic intra-articular environment and oxidative stress induce the production of pro-inflammatory cytokines (106). Ng and coll. confirmed this link by demonstrating a high expression of NOX in RA and PsA synovial tissue, which correlates with intra-articular pO2 and angiogenesis. A reduction of NOX expression was observed after 3 months of anti-TNFα treatment, in association with raised pO2 and a lower disease activity (123). The oxidative stress, measured as rate of mitochondrial DNA (mtDNA) mutations, is increased in RA and PsA synoviocytes; and it is correlated to high reactive ROS, reduced expression of cytochrome c oxidase, and low level of intra-articular pO2 (8). As a further evidence of the link between inflammation and oxidative stress, Harty et al demonstrated that the levels of mitochondrial DNA (mtDNA) point mutations in synovial tissue from patients with inflammatory arthritis was related to *in vivo* hypoxia and oxidative stress levels (124). The authors described a correlation between mtDNA mutation rate, the expression of TNFα and macroscopic arthroscopic signs of inflammation in RA and PsA patients. Patients were then treated with anti-TNFα, and only in anti-TNFα-responders the mtDNA mutations frequency was reduced. Moreover, the addition of recombinant TNFα in RA and PsA synoviocytes culture induces mtDNA mutations (124). These data suggest that antioxidant agents may be potentially useful as an add-on treatment to conventional therapies in the management of inflammatory arthritis and in autoimmune diseases (125, 126).

## Conclusions

All studies converge to establish that in autoimmune diseases as PsO and PsA, oxidative stress, cell apoptosis and inflammation may lead to cytochrome c released from the mitochondria into the extracellular space. The future perspective given by data from the literature is that serum cytochrome c can be measured and used for diagnosing and assessing cell death during systemic diseases. The role of cytochrome c has been associated with autoimmune diseases and its release from mitochondria into the cytoplasm may be considered as a marker of inflammation and autoimmunity. Besides, several evidence suggests that mast cells activation is implicated in PsO and PsA pathogenesis. Those cells and the release of tryptase support the relevant role of innate immunity in PsO and PsA development. In this context, both cytochrome c and tryptase are detected during the inflammatory process. They may act as potential triggers in the perpetuation of the pro-inflammatory loop. In this context, mediators released from mast cells or mitochondrial activity may be used as marker of disease or as target in the treatment of autoimmune diseases. However, further studies are needed to better understand the potential contribution of cytochrome c and tryptase in autoimmune disease pathogenesis during oxidative stress and their potential correlation with disease activity and as markers of treatment efficacy.

## Author contributions

MC, FS, and LF conceived the review and wrote the introduction and the paragraph concerning psoriatic arthritis. GF, ME, AG, and RS helped in writing the part concerning cytochrome c and tryptase. EB and LB wrote the paragraph concerning psoriasis. PC, PT, FC, and LC supervised the paper. RP conceived and revised the manuscript.

### Conflict of interest statement

The authors declare that the research was conducted in the absence of any commercial or financial relationships that could be construed as a potential conflict of interest.
